# The association between PD-L1 and EGFR status and the prognostic value of PD-L1 in advanced non-small cell lung cancer patients treated with EGFR-TKIs

**DOI:** 10.18632/oncotarget.3694

**Published:** 2015-03-29

**Authors:** Yanna Tang, Wenfeng Fang, Yaxiong Zhang, Shaodong Hong, Shiyang Kang, Yue Yan, Nan Chen, Jianhua Zhan, Xiaobo He, Tao Qin, Ge Li, Wenyi Tang, Peijian Peng, Li Zhang

**Affiliations:** ^1^ Department of Medical Oncology, Sun Yat-sen University Cancer Center, Guangzhou, China; ^2^ State Key Laboratory of Oncology in South China, Guangzhou, China; ^3^ Collaborative Innovation Center for Cancer Medicine, Guangzhou, China; ^4^ Key Laboratory for Stem Cells and Tissue Engineering, Sun Yat-sen University, Guangzhou, China; ^5^ Department of Medical Oncology, The Fifth Affiliated Hospital of Sun Yat-Sen University, Zhu Hai, China

**Keywords:** NSCLC, PD-L1, EGFR status, TKI, prognosis

## Abstract

**Backgrounds:**

Recent clinical trials have shown that immune-checkpoint blockade yields remarkable response in a subset of non–small cell lung cancer (NSCLC) patients. However, few studies directly focus on the association between epidermal growth factor receptor (EGFR) mutational status and programmed cell death-ligand 1 (PD-L1) expression. We examined whether PD-L1 is related to clinicopathologic factors and prognosis in patients with advanced NSCLC treated with EGFR-tyrosine kinase inhibitors (EGFR-TKIs).

**Methods:**

One-hundred and seventy patients with advanced NSCLC were explored. Paraffin-embedded tumour sections were stained with PD-L1 antibody. EGFR mutation was examined by fluorescent quantitative polymerase chain reaction (PCR). The correlations between PD-L1 expression and EGFR status and survival parameters were analyzed.

**Results:**

The overall frequency of PD-L1 over-expression was 65.9% (112/170). In lung adenocarcinoma, PD-L1 tended to be associated with mutant EGFR (PD-L1 overexpression in mutant and wild-type EGFR, 64/89 (71.9%) vs. 32/56 (57.1%), respectively; p=0.067). Subgroup analyses showed that high PD-L1 expression was associated with significantly shorter overall survival (OS) in EGFR wild-type patients (p=0.029) but not in EGFR mutant patients (p=0.932) treated with EGFR-TKIs. Even more, for EGFR mutant patients, higher expression of PD-L1 might only signal better outcome with TKIs.

**Conclusions:**

High PD-L1 expression was likely to be associated with the presence of EGFR mutation in advanced lung adenocarcinoma. For EGFR wild-type patients, the PD-L1 over expression can be considered as a poor prognostic indicator of OS.

## INTRODUCTION

Lung cancer, especially non-small cell lung cancer (NSCLC), is currently the leading cause of cancer-related death worldwide [1]. Recent advancements in targeted therapy have led to a major paradigm shift in the treatment of advanced NSCLC [2]. Molecularly targeted drugs such as erlotinib and gefitinib have thus greatly improved the clinical outcome of advanced NSCLC patients harboring sensitive epidermal growth factor receptor (EGFR) gene mutations [3]. Two major types of EGFR kinase mutations include exon-19 deletions and L858R mutation in exon 21 [4, 5].

Programmed death 1 (PD-1) is a co-inhibitory receptor expressed on the membrane of activated T and B cells [6], which plays a crucial role in tumor immune escape [7, 8]. Programmed cell death-ligand 1 (PD-L1) is the major ligand for PD-1 and is expressed in a variety of cancers [7, 9]. PD-L1 has been shown to be involved in the negative regulation of immune response through PD-1 receptor and has been thought to be an important strategy for cancer cells to evade host immune surveillance. Cancer cells expressing PD-L1 have been shown to increase apoptosis of antigen-specific human T-cell clones and to inhibit CD4 and CD8 T-cell activation *in vitro* [10-12].

Currently, some studies demonstrated that PD-L1 was expressed in 19.63%-65.38% of NSCLC [2, 13-16]. Several studies suggested that PD-L1 expression portended inconsistent survival outcomes [17]. For example, a study showed that tumor with a high level of PD-L1 expression was associated with significantly shorter overall survival (OS) in NSCLC patients [2], while another report showed positive PD-L1 was significantly associated with better survival outcome [15]. Now, the molecular regulatory mechanism of PD-L1 isn't comprehensive enough, though two studies found that mutant EGFR could induce PD-L1 expression *in vitro* and *vivo*, and EGFR tyrosine kinase inhibitors (EGFR-TKIs) could down-regulate PD-L1 expression [2, 16]. It is therefore of significance to study the association between EGFR driver mutation and PD-L1. Even more, the incidence of EGFR mutations is higher in East Asian patients than in Caucasian patients (30% versus 8%) [4], and there are no studies examining the relationship between PD-L1 expression and efficacy in patients treated with EGFR-TKIs in China. Therefore, we tried to investigate the impact of PD-L1 expression on EGFR-TKIs' efficacy and prognosis in Chinese advanced NSCLC patients, emphasizing on the EGFR mutational status.

## RESULTS

### Patient characteristics

**Figure 1:**
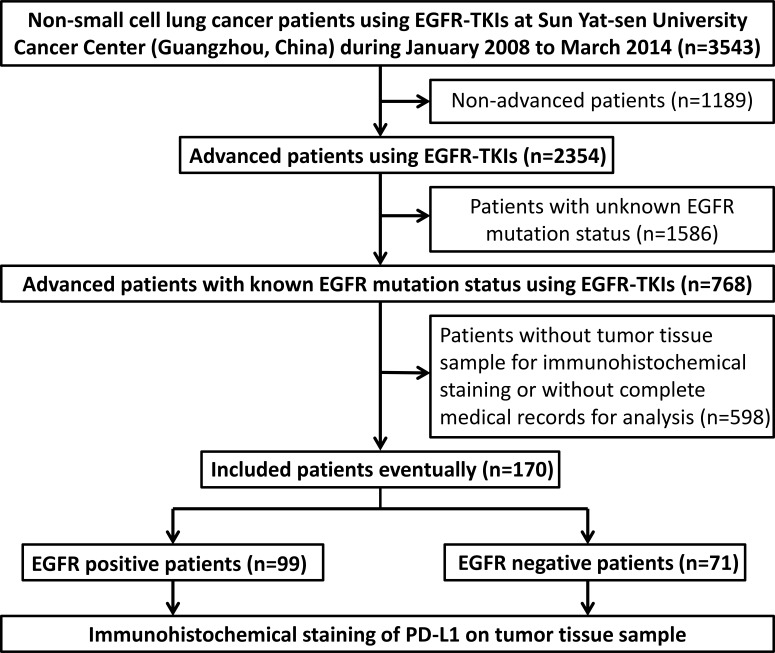
Flowing chart of the enrollment Abbreviations: EGFR, epidermal growth factor receptor; EGFR-TKIs, EGFR-tyrosine kinase inhibitors; PD-L1, programmed cell death-ligand 1.

A total of 170 eligible patients with advanced NSCLC were included in the present study (Figure 1). The mean age at diagnosis was 57.09 years (range, 32–80 years) (Table 1). Seventy-seven (45.3%) of the patients were female and ninety-three (54.7%) were male. Fifty-seven (33.5%) patients were smokers. Nine (5.3%) patients and 161 (94.7%) patients were diagnosed at stage IIIB and stage IV, respectively. In the current research, 99 patients harbored EGFR mutation and the remaining 71 patients were EGFR wild type. With regard to EGFR mutation status, 40 patients harbored a deletion in exon 19 and 47 patients had an L858R missense mutation in exon 21. 12 were uncommon mutation.

**Table 1:** Clinicopathological characteristics of patients with NSCLC (n = 170) and their relationship with PD-L1 expression

Parameter	PD-L1 positive				PD-L1 negative				
	total	PR	SD	PD	total	PR	SD	PD	P-value
**Age**									
≤50y	28	6	17	5	17	3	9	5	0.546
>50y	84	19	51	14	41	12	22	7	
**Gender**									
female	56	13	38	5	21	8	11	2	0.087
male	56	12	30	14	37	7	20	10	
**Smoking**									
no	77	21	50	6	36	14	15	7	0.382
yes	35	4	18	13	22	1	16	5	
**Pathology**									
ADC	96	24	58	14	49	14	25	10	0.830
non-ADC	16	1	10	5	9	1	6	2	
**Stage**									
III B	7	1	5	1	2	0	2	0	0.680
IV	105	24	63	18	56	15	29	12	
**EGFR status**									
wild type	42	5	21	16	29	3	16	10	0.330
exon 19 del	26	6	19	1	14	7	6	1	
exon 21 L858R	35	11	23	1	12	4	7	1	
unknown classical	9	3	5	1	3	1	2	0	
**EGFR-TKI**									
1st line	61	16	37	8	22	8	12	2	**0.041**
≥2nd line	51	9	31	11	36	7	19	10	
**Pathology=ADC**									
EGFR wild type	32	4	15	13	24	3	13	8	0.067
EGFR mutation	64	20	43	1	25	11	12	2	

Notes: H-score ≥ 5 was used as cut-off value to distinguish positive and negative expression of PD-L1.

Abbreviations: PD-L1, programmed cell death-ligand 1; ADC, adenocarcinoma; non-ADC, non-adenocarcinoma; EGFR, epidermal growth factor receptor; EGFR-TKIs, EGFR-tyrosine kinase inhibitors.

### Correlations between PD-L1 expression and baseline characteristics

**Figure 2:**
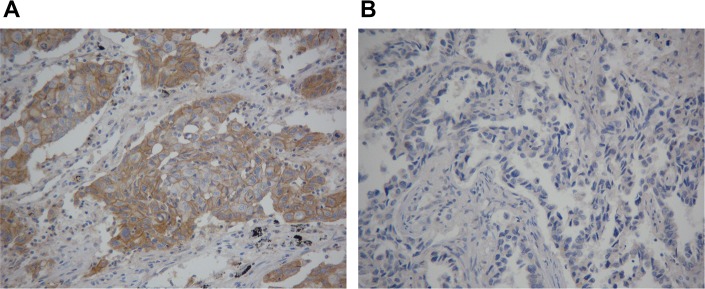
(**A**) Positive programmed cell death-ligand 1 (PD-L1) immunohistochemical staining with a membranous pattern. (**B**) Negative PD-L1 immunohistochemical staining. Original magnification, 20 ×.

Immunohistochemical staining for PD-L1 was found at the membrane or in the cytoplasm (or both) of tumor cells (Figure 2A). As shown in Table 1, PD-L1 is over expressed in 65.9% (112/170) of advanced NSCLC patients. The relationship between PD-L1 expression and age, gender, histopathological type, tumor stage and EGFR mutational status was not significant, except for the line of EGFR TKIs (*P* = 0.041). However, in subgroup of lung adenocarcinoma, there was a borderline difference between PD-L1 expression level and EGFR mutational status (32/56 (57.1%) for wild type and 64/89 (71.9%) for mutant type, respectively, p=0.067).

### Relationships between PD-L1 expression and the EGFR-TKIs' efficacy

The association between the efficacy of EGFR-TKIs with PD-L1 expression as well as other clinicpathologic factors in advanced NSCLC patients was summarized in Table 2. There was no significant relationship between objective response rate (ORR) and PD-L1 expression, as well as age, gender, histopathological type, stage and TKI line. However, patients with mutant EGFR had better ORR than those with wild-type EGFR (odds ratio (OR), 0.266; 95% confidence interval (95%CI), 0.114 to 0.621; p =0.002) and non-smokers also had higher ORR than smokers did (OR, 4.667; 95% CI, 1.716 to 12.693; p = 0.003). These results were in accordance with the results of multivariate analysis. Besides, we examined the association between a variety of factors and disease control rate (DCR). We found that there was no significant difference between DCR and PD-L1 status (OR, 0.783; 95% CI, 0.350 to 1.751; p =0.551). Whereas, DCR was significantly higher in women than that in men (OR, 3.478; 95% CI, 1.407 to 8.600; P=0.007), in never-smokers than that in smokers (OR, 3.55; 95% CI, 1.589 to 7.930; P=0.002), and in those with EGFR mutation than that in those EGFR with wild type (OR, 0.092; 95% CI, 0.033 to 0.256; P<0.001) (Table 2). And the multivariate analysis revealed that EGFR mutation positivity was an independent factor (OR, 0.113; 95% CI, 0.038 to 0.342; P=0.007). We further divided patients into two subgroups: (I) EGFR wild type (n=71) and (II) EGFR mutant (n=99). No significant differences in two subgroups were found between PD-L1 expression and ORR (OR, 0.854; 95% CI, 0.187 to 3.891; P=0.838 and OR, 1.765; 95% CI, 0.715 to 4.353; P=0.218 for group I and group II, respectively), as well as PD-L1 expression and DCR (OR, 1.169; 95% CI, 0.436 to 3.137; P=0.756 and OR, 0.604; 95% CI, 0.096 to 3.822; P=0.593 for group I and group II, respectively).

**Table 2:** The association between PD-L1 expression and EGFR-TKIS' efficacy in univariate and multivariate logistic regression analysis#

Parameter	ORR									DCR								
		Univariate analysis						Multivariate analysis			Univariate analysis						Multivariate analysis		
			PR	SD+PD	OR	95%CI	P-value	OR	95%CI	P-value	PD	PR+SD	OR	95%CI	P-value	OR	95%CI	P-value
**Age**																		
≤50		9	36	0.758	0.329-1.748	0.516	0.663	0.264-1.666	0.382		10	35	0.707	0.304-1.645	0.421	0.495	0.181-1.358	0.172
>50	1	31	94							1	21	104						
**Gender**																		
female		21	56	1.461	0.717-2.974	0.296	0.541	0.220-1.331	0.181		7	70	3.478	1.407-8.600	**0.007**	1.899	0.528-6.837	0.326
male	1	19	74							1	24	69						
**Smoking**																		
no		35	78	4.667	1.716-12.693	**0.003**	5.776	1.763-18.863	**0.004**		13	100	3.55	1.589-7.930	**0.002**	1.942	0.612-6.154	0.260
yes	1	5	52							1	18	39						
**Pathology**																		
ADC		38	107	4.084	0.919-18.150	0.064	3.834	0.793-18.538	0.095		24	121	1.961	0.738-5.207	0.177	1.348	0.439-4.142	0.602
non-ADC	1	2	23							1	7	18						
**Stage**																		
IIIB		1	8	0.391	0.047-3.225	0.383	0.206	0.023-1.877	0.161		1	8	1.832	0.221-15.208	0.575	0.576	0.046-7.175	0.668
IV	1	39	122							1	30	131						
**EGFR**																		
wild type		8	63	0.266	0.114-0.621	**0.002**	0.351	0.137-0.904	**0.030**		26	42	0.092	0.033-0.256	**<0.001**	0.113	0.038-0.342	**<0.001**
mutation	1	32	67							1	5	97						
**EGFR-TKI**																		
1st line		24	59	1.805	0.878-3.711	0.108	1.863	0.798-4.349	0.150		10	73	2.323	1.020-5.291	**0.045**	1.394	0.525-3.702	0.506
≥2nd line	1	16	71							1	21	66						
**PD-L1**																		
negative		15	43	1.214	0.581-2.537	0.606	1.674	0.711-3.939	0.238		12	46	0.783	0.350-1.751	0.551	1.109	0.445-2.764	0.824
positive	1	25	87							1	19	93						
**EGFR wild-type subgroup**																		
PD-L1 negative		3	26	0.854	0.187-3.891	0.838					10	19	1.169	0.436-3.137	0.756			
PD-L1 positive	1	5	37							1	16	26						
**EGFR mutant subgroup**																		
PD-L1 negative		12	17	1.765	0.715-4.353	0.218					2	27	0.604	0.096-3.822	0.593			
PD-L1 positive	1	20	50							1	3	67						

# A total of 170 non-small cell lung cancer patients were included.

Abbreviations: ORR, objective response rate; DCR, disease control rate; PR, partial response; SD, stable disease; PD, progressive disease ; OR, odd ratio; 95%CI, 95% confidence intervals; ADC, adenocarcinoma; non-ADC, non-adenocarcinoma; EGFR, epidermal growth factor receptor; EGFR-TKI, EGFR-tyrosine kinase inhibitor; PD-L1, programmed cell death-ligand 1.

### Survival analyses is in NSCLC patients

**Figure 3:**
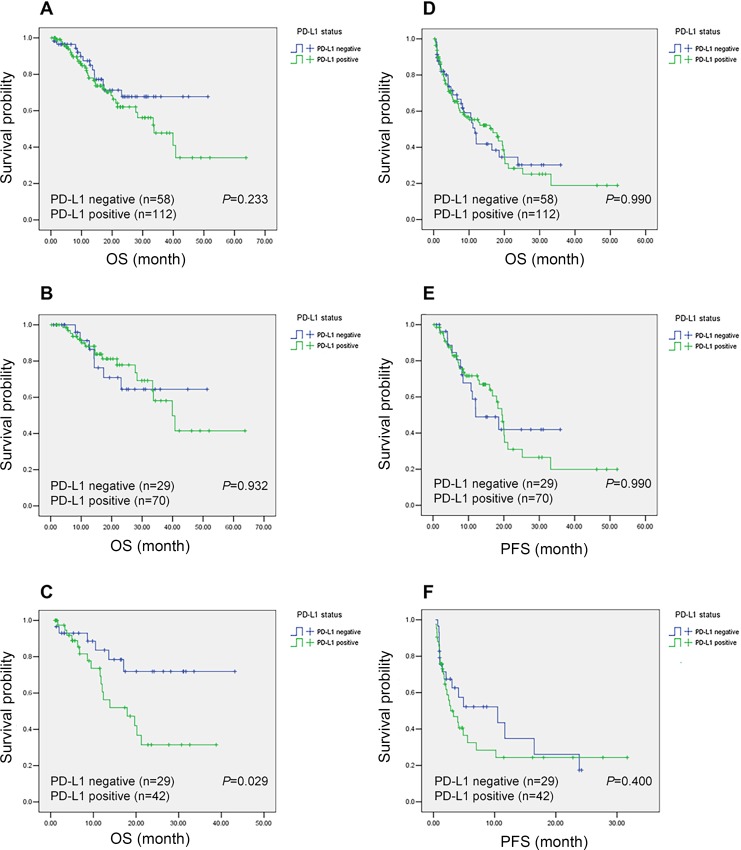
Kaplan-Meier curves of overall survival (OS) and progression-free survival (PFS) according to programmed cell death-ligand 1 (PD-L1) expression status in non-small cell lung cancer (NSCLC) patients The P value for the difference between the two curves was determined by the log-rank test. Notes: (**A**) OS for overall population. (**B**) OS for patients with EGFR mutation. (**C**) OS for patients with EGFR wild type. (**D**) PFS for overall population. (**E**) PFS for patients with EGFR mutation. (**F**) PFS for patients with EGFR wild type.

**Figure 4:**
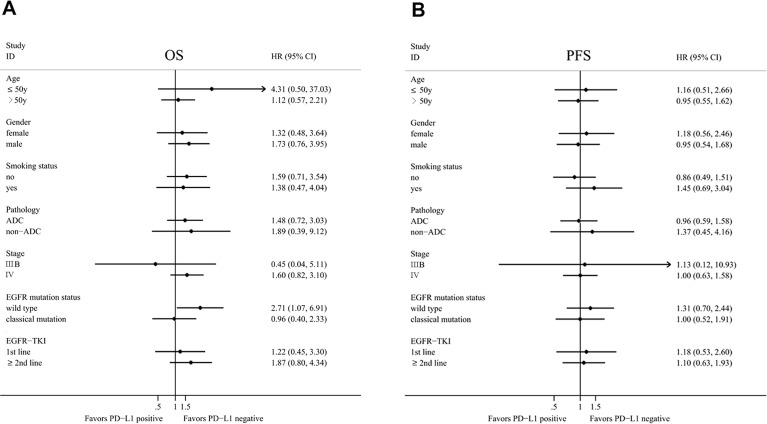
Forest plot of overall survival (OS) and progression-free survival (PFS) according to programmed cell death-ligand 1 (PD-L1) expression status in subgroup analysis Abbreviations: HR, hazard ratio, HR <1 implies a lower risk of progression or death for patients; 95% CI, 95% confidence intervals.

The median overall survival of the whole patients was 39.9 months. Kaplan–Meier analysis revealed that overall patients with positive PD-L1 and negative PD-L1 expression had no significant difference in OS and progression-free survival (PFS) (Figure 3A and 3D). To investigate the PD-L1's clinical significance, we further divided the patients into two groups: EGFR mutation and EGFR wild type. In EGFR mutation group, PFS and OS of patients with positive PD-L1 tended to be longer than patients with negative PD-L1, although statistical significance was not achieved (Figure 3B and 3E). In EGFR wild type group, negative PD-L1 patients have longer OS than positive-PD-L1 patients (P=0.029) (Figure 3C), while no significant difference in PFS was observed (Figure 3F). Exploratory analysis was done to validate the prognostic role of PD-L1 in subgroups defined by age, sex, smoking, pathology, stage of disease, EGFR mutation, and EGFR-TKI's lines (Figure 4). We found that patients with positive PD-L1 expression had signal better prognosis in EGFR mutation subgroup, contrary to EGFR wild-type group. To determine the prognostic value of PD-L1 expression, we carried out univariate and multivariate analyses using the Cox regression model. For PFS and OS, EGFR mutation remained the independent factor for better prognosis (hazard ratio (HR), 0.419; 95% CI, 0.262-0.672; P <0.001 for PFS and HR, 0.499; 95% CI, 0.264-0.942; P=0.032 for OS, respectively) (Table 3). In subgroup multivariate analysis, we found that the high level PD-L1 can be considered as a poor prognostic indicator of OS for EGFR wild-type patients (HR, 3.738; 95% CI, 1.341-10.419; P=0.012) (Supplementary table 1).

**Table 3:** Univariate and multivariate analyses of OS and PFS in NSCLC patients

Parameter	OS								PFS							
	Univariate analysis					Multivariate analysis			Univariate analysis					Multivariate analysis		
		N	HR	95%CI	P-value	HR	95%CI	P-value		N	HR	95%CI	P-value	HR	95%CI	P-value
**Age**																
≤50y	1	45							1	45						
>50y		125	2.552	1.082-6.019	**0.032**	2.602	1.078-6.279	**0.033**		125	0.77	0.483-1.229	0.274	0.69	0.428-1.112	0.128
**Gender**																
female	1	77							1	77						
male		93	1.737	0.963-3.131	0.066	1.346	0.649-2.795	0.425		93	1.779	1.147-2.760	**0.010**	1.245	0.704-2.200	0.451
**Smoking**																
no	1	113							1	113						
yes		57	1.718	0.921-3.207	0.089	1.218	0.552-2.684	0.626		57	1.832	1.179-2.846	**0.007**	1.364	0.761-2.445	0.297
**Pathology**																
ADC	1	145							1	145						
non-ADC		25	1.349	0.604-3.017	0.465	1.548	0.680-3.520	0.298		25	1.59	0.894-2.826	0.114	1.396	0.776-2.508	0.265
**Stage**																
IIIB	1	9							1	9						
IV		161	1.199	0.370-3.890	0.762	1.030	0.291-3.650	0.963		161	1.627	0.594-4.455	0.344	0.822	0.284-2.382	0.719
**EGFR**																
wild type	1	71							1	71						
mutation		99	0.485	0.271-0.868	**0.015**	0.499	0.264-0.942	**0.032**		99	0.365	0.237-0.562	**<0.001**	0.419	0.262-0.672	**<0.001**
**EGFR-TKI**																
1st line	1	83							1	83						
≥2nd line		87	1.338	0.751-2.384	0.323	1.562	0.839-2.906	0.159		87	1.941	1.257-2.996	**0.003**	1.756	1.099-2.806	**0.019**
**PD-L1**																
negative	1	58							1	58						
positive		112	1.472	0.776-2.792	0.236	1.901	0.953-3.790	0.068		112	1.003	0.641-1.569	0.990	1.315	0.831-2.080	0.242

Abbreviations: OS, overall survival; PFS, progression-free survival; HR, hazard ratio; 95%CI, 95% confidence intervals; ADC, adenocarcinoma; non-ADC, non-adenocarcinoma; EGFR, epidermal growth factor receptor; EGFR-TKI, EGFR-tyrosine kinase inhibitor; PD-L1, programmed cell death-ligand 1.

## DISCUSSION

NSCLC is partially characterized by driver mutation-defined molecular subsets, each with distinct clinicopathologic features and potentials for targeted therapies. In the present study, we found that PD-L1 was over-expressed in 65.9% of advanced NSCLC samples and positive PD-L1 tended to be associated with EGFR mutation. We also revealed that there was no significant correlation between expression of PD-L1 and curative effect of EGFR TKIs (ORR and DCR). In EGFR mutation group, PFS and OS of patients positive for PD-L1 tended to be signal better than that of patients who were negative, although statistical significance was not achieved. For patients with wild type EGFR, PD-L1-negative NSCLC patients had longer overall survival than PD-L1-positive ones.

In previous studies, researchers have demonstrated that activation of the EGFR pathway induced PD-L1 expression [2, 16, 18], and found PD-L1 was significantly higher in patients with the following characteristics: women, never smokers and with adenocarcinoma [2, 16]. All these characteristics are hallmarks of EGFR mutations. In present study, we also found a borderline association between high PD-L1 expression and EGFR mutation in lung adenocarcinoma, while no significant relationship between ages, gender, smoking history and PD-L1 expression was found in NSCLC patients. The difference between the present and previous studies may be due to several reasons. First, the sample size varied among different studies and all data were retrospectively collected, resulting in potential bias. Second, the heterogeneity of baseline characteristics among these studies had also affected their outcomes, such as pathological stage and geographical distribution. Third, the threshold of positive PD-L1 expression was also different from each other in these studies. Fourth, the specificity and reproducibility of the commercially available antibodies and variations in Immunohistochemical technique weren't thoroughly assessed [15]. Thus, for future studies, more efforts to standardize a quantitative assay for PD-L1 expression are warranted.

In EGFR wild-type lung cancer, immune evasion induced by PD-L1 played an important role. PD-1/PD-L1 pathway has been recognized as a key mechanism of immune evasion. Cancer cells can evade host immune systems by expressing certain ligands to down-regulate cytotoxic T lymphocytes through inhibitory pathways, which are usually initiated by ligand-receptor interactions [19]. PD-1 is one immune checkpoint expressed on the surface of T-cells upon activation [20]. PD-L1 is the major ligand for PD-1 and is expressed in various type of cancers [9]. PD-1/PD-L1 interaction is regarded as an inhibitory checkpoint for T-cell activation at its initial stage. In the present report, our data revealed that high PD-L1 expression was correlated with poor prognosis in EGFR wild-type patients but not in EGFR mutant population. This finding indicated that EGFR wild-type NSCLC failed to be controlled by the immune system which is inhibited by PD-L1 mediated antitumor activity. Then cancer cells can evade host immune responses by expressing PD-L1 to down-regulate T-cell activation in tumorous microenvironment [9], allowing cancer cells to survive and progress. Therefore, PD-L1 status was a significant prognostic factor for patients with EGFR wild type. This implicated that for this subset of population (EGFR wild-type and PD-L1 over-expression), PD-L1 blocker may be an alternative therapeutic strategy. For future clinical applications, more evidences to verify the feasibility are warranted.

According to previous studies, we could explain why patients with positive for PD-L1 expression tended to have better OS than PD-L1 negative ones in EGFR mutant NSCLC patients. The most possible reason was the cross interaction between EGFR pathway and PD-L1. A study by Azuma K et al. [2] found that inhibition of EGFR signaling with erlotinib led to down-regulation of the expression of PD-L1 in EGFR mutant NSCLC cells but not in those with wild-type EGFR, indicating that the expression of PD-L1 might be dependent on EGFR signaling conferred by activating EGFR mutations. In another research, Akbay EA et al. [16] also found that PD-L1 expression was reduced by EGFR inhibitors in NSCLC cell lines with activated EGFR. Therefore, for EGFR mutant NSCLC patients, EGFR-TKIs could perform a dual therapeutic response. The down-regulation of PD-L1 expression and the consequent restoration of an antitumor immune response might contribute to the durable therapeutic response. EGFR activation up regulated PD-L1 through MAPK signaling pathway. As previously reported, EGFR remarkably increased the activity of ERK1/2 and AKT which are involved in the proliferation, anti-apoptosis, and invasion of tumor cells [21, 22]. Chen N et al. [23] further demonstrated that the up-regulation of PD-L1 mediated by EGFR activation was associated with the activation of ERK1/2/c-Jun. By inhibiting p-ERK1/2, PD-L1 decreased following p-ERK1/2/p-Jun down-regulation in a clear dose-dependent manner [23]. This detailed mechanism confirmed that EGFR mutant NSCLC patients may benefit not only from direct tumor killing effect of EGFR-TKIs but also indirectly from immune enhancement after EGFR-TKIs treatment.

As we known, targeted therapy usually have rapid and impressive response rates but modest progression-free survival while immunotherapy can achieve durable tumor control but associated with lower response rates [24]. To address this, investigators have proposed combining these strategies. There is a scientific rationale supporting the combination of targeted therapy and immunotherapy [25]. For patients with melanoma, the concept of potential synergy with BRAF-targeted therapy and immunotherapy is being empirically investigated in clinical trials [26-28]; however, much remains to be learned. Response data from these initial trials are not mature, and additional trials will be needed to determine the appropriate sequence, schedule, and duration of therapy if there is evidence of synergy. Yet, there were no data about the combinatorial strategies of EGFR-TKIs with anti-PD-1/PD-L1. While there were no direct verified evidences, this combining therapy may be an alternative strategy in EGFR mutant NSCLC. In the future, more well-designed *in vitro* and *vivo* studies to explore molecular mechanisms of combining EGFR-TKIs and anti-PD-1/PD-L1 antibodies are urgently required. Randomized clinical trials to instruct how best to combine therapeutic agents are also needed.

Currently, though gefitinib and erlotinib are regarded as the first line treatment of classical EGFR mutant NSCLC patients, a majority of them eventually develop secondary resistance to gefitinib and erlotinib. Previous treatment options for EGFR-TKIs resistance include CO-1686 [29], AZD9291 [30] and HM61713 [31] for EGFR T790M and EGFR-TKIs plus c-met inhibitors for c-met amplification [32]. However, the role of immunotherapy in EGFR-TKIs-resistant patients has not been revealed. Chen N et al. [23] demonstrated that the protein level of PD-L1 in EGFR-mutant NSCLC cell lines (PC-9, HCC827 and H1975) was significantly higher than that in EGFR-wild type cell lines (A539, H1993). Moreover, the expression of PD-L1 was the highest in resistant cells (H1975 cells, with EGFR-T790M mutation). Anti- PD-1/PD-L1 axis could significantly decrease the viability of gefitinib resistant H1975 cells. This implied that blockade of PD-1/PD-L1 might be a promising optional treatment for NSCLC patients with EGFR mutation, especially for EGFR-TKIs resistant NSCLC patients. Future clinical studies are needed to test the feasibility.

Notably, this is the first study to assess the relationship between PD-L1 expression and prognosis as well as efficacy in Chinese advanced NSCLC patients with treatment of EGFR-TKIs. We prove that high PD-L1 expression is likely to be associated with the presence of EGFR mutation in advanced lung adenocarcinoma. Moreover, PD-L1 over-expression can be considered as a poor prognostic indicator of OS in EGFR wild-type patients treated with EGFR-TKIs.

## PATIENTS AND METHODS

### Patients

A total of 3543 consecutive NSCLC patients who have taken oral EGFR-TKIs at Sun Yat-sen University Cancer Center (Guangzhou, China) from January 2008 to March 2014 were screened in the study. Patients were recruited if they met the following conditions: 1, treated with EGFR-TKIs; 2, stage 3b/4 NSCLC or recurrent disease after surgery or chemotherapy; 3, with known EGFR mutational status; 4, having detailed medical records and had enough tumor tissue samples for immunohistochemical staining of PD-L1. Finally, a total of 170 patients were eligible. Figure 1 summarized the process of patients' selection. Baseline clinical and pathological features were collected, as well as tissue specimens from surgery or biopsy. The clinicopathological features of the patients included age, gender, smoking status, pathological type, Union for International Cancer Control (UICC) stage (the seventh edition), EGFR mutation status and EGFR-TKI treatment history. Fifty years old was chosen as the cutoff for dividing the high or low age group (50y was low and 50y was high, respectively). Smoking history was noted as yes or no (no-smoking refers to patients who had never smoked in their lifetime). Pathological subtype was divided into adenocarcinoma or non-adenocarcinoma. All patients were restaged according to the seventh edition of UICC Staging System for NSCLC. EGFR exon 19 deletions or exon 21 base substitutions were considered as classical EGFR mutations by fluorescent quantitative polymerase chain reaction (PCR). The study was approved by the Institutional Review Board of Sun Yat-Sen University Cancer Center (Guangzhou, China). All the patients had provided written informed consent before samples were collected.

### Immunohistochemistry analyses

Immunohistochemical staining was performed using rabbit monoclonal anti-human antibody (E1L3N™, Cell Signaling Technology, Danvers, MA, 1:200) for testing the expression of PD-L1 in human NSCLC specimens. Five-μm-thick Sections were cut from the formalin-fixed, paraffin-embedded (FFPE) tumor block and then routinely deparaffined and rehydrated. For antigen retrieval, slides were heated in a microwave oven for 30 minutes in citrate buffer solution (pH=7.4) and cooled slowly at room temperature for 20 minutes. After blocking the activity of endogenous peroxidase with 3% hydrogen peroxide for 8 minutes, the sections were treated with primary antibodies and incubated for overnight (more than 12 hours). Subsequently, the slides were rinsed in PBS three times and incubated in HRR-linked secondary antibodies. After incubation, slides were washed again with PBS and then visualized using diaminobenzidine. Finally, Mayer's hematoxylin was used to counterstain the sections, which were then dehydrated and mounted.

Two pathologists who were blinded to the clinical or pathological information of these patients independently assess the expression of PD-L1. Semiquantitative H score (maximum value of 300 corresponding to 100% of tumor cells positive for PD-L1 with an overall staining intensity score of 3) was defined as multiplying the percentage of stained cells by an intensity score (0, absent; 1, weak; 2, moderate; and 3, strong). A 5% proportion of membrane-positive tumor cells which were defined as H-score ≥ 5 have been used as cutoff for PD-L1 positivity [33, 34], as this cut-point is reported to be associated with clinical response to anti-PD-1 therapy [34].

### Statistical analysis

All the statistical analysis was performed using SPSS 20.0 for Windows (IBM, Armonk, NY). The cut-off value of age were obtained by X-tile software (Version 3.6.1, Yale University, New Haven, CT), taking clinical expertise into consideration. Pearson's chi-squared test or continuity correction test was used to assess correlations between PD-L1 expression and clinicopathologic variables. Univariate and multivariate logistic regression analysis were used to test the association between PD-L1 expression and EGFR-TKIs' efficacy (ORR and DCR). OS was defined as the time from diagnosis to the end of the follow-up (August 2014). And PFS was the time from beginning to taking TKI to recurrence or last follow-up. OS and PFS analyses were estimated with Kaplan-Meier method and multivariable analyses were performed to assess survival difference. The results of ORR and DCR were reported with OR and its 95% CI, while prognostic results were reported with HR and its 95% CI. OR > 1 indicated that EGFR-TKI was more effective in PD-L1 positive patients. HR < 1 implied a lower risk of progression or death for patients with positive expression of PD-L1. A two sided p-value of <0.05 was considered statistically significant.

## SUPPLEMENTARY MATERIAL TABLE


