# Supplementary material for: The association between PD-L1 and EGFR status and the prognostic value of PD-L1 in advanced non-small cell lung cancer patients treated with EGFR-TKIs
Source: Oncotarget. 2015 Mar 29;6(16):14209–19. doi: 10.18632/oncotarget.3694 (PMC4546461; doi:10.18632/oncotarget.3694)
Supplement: Supplementary file 1 [file oncotarget-06-14209-s001.pdf]

The association between PD-L1 and EGFR status and the prognostic value of PD-L1 in advanced non-small cell lung cancer patients treated with EGFR-TKIs

Supplementary Material

Supplementary Table 1: Multivariate analyses of OS in two subgroups

Parameter	EGFR wild type Multivariate analysis					EGFR mutation Multivariate analysis				
		N	HR	95%CI	P-value		N	HR	95%CI	P-value
**Age**										
≤50y	1	18				1	27			
>50y		53	1.449	0.423-4.964	0.555		72	3.667	0.810-16.605	0.092
**Gender**										
female	1	23				1	54			
male		48	1.244	0.385-4.017	0.715		45	1.413	0.529-3.776	0.491
**Smoking**										
no	1	37				1	76			
yes		34	1.867	0.600-5.804	0.281		23	1.130	0.294-4.343	1.130
**Pathology**										
ADC	1	56				1	89			
non-ADC		15	1.159	0.308-4.367	0.827		10	1.897	0.604-5.956	0.273
**Stage**										
IIIB	1	1				1	8			
IV		70	0.703	0.081-6.131	0.750		91	1.500	0.313-7.185	0.612
**EGFR-TKI**										
1st line	1	22				1	61			
≥2nd line		49	2.200	0.836-5.790	0.110		38	1.208	0.504-2.896	0.672
**PD-L1**										
negative	1	29				1	29			
positive		42	3.738	1.341-10.419	**0.012**		70	0.888	0.356-2.215	0.799

Abbreviations: OS, overall survival; PFS, progression-free survival; HR, hazard ratio; 95%CI, 95% confidence intervals; ADC, adenocarcinoma; non-ADC, non-adenocarcinoma; EGFR, epidermal growth factor receptor; EGFR-TKI, EGFR-tyrosine kinase inhibitor; PD-L1, programmed cell death-ligand 1.
